# Effects of Escin on Oxidative Stress and Apoptosis of H9c2 Cells Induced by H_2_O_2_

**DOI:** 10.1155/2022/7765353

**Published:** 2022-01-27

**Authors:** Peng Qiao, Baokun Zhang, Xueni Liu, Jie Xu, Xuehan Li

**Affiliations:** ^1^Department of Traditional Chinese Medicine, Yantaishan Hospital, Yantai, China; ^2^Public Health, The University of Sheffield, UK; ^3^Critical Care Medicine, PLA Rocket Force Characteristic Medical Center, Beijing, China; ^4^Department of Medical Security Center, PLA Rocket Force Characteristic Medical Center, Beijing, China; ^5^Department of Geriatric, Liaocheng People's Hospital, Liaocheng, China

## Abstract

**Objective:**

Myocardial infarction (MI) is a serious heart health problem in the world with a high mortality rate. Our study is mainly aimed at validating the antioxidative stress and antiapoptotic effects of escin in a H_2_O_2_-induced cardiomyocyte injury model.

**Methods:**

H9c2 cells were divided into control group, H_2_O_2_ treatment group, and H_2_O_2_+escin group. We studied the effect of escin on H9c2 cells and its mechanism by flow cytometry, real-time PCR, CCK-8 assay and Western blot. Cell morphology was observed by cell staining and optical microscopy.

**Results:**

We found that the level of reactive oxygen species (ROS) in the H_2_O_2_ treatment group was significantly elevated, while the high level of ROS was significantly reversed after treatment with escin. The protein levels of SOD1, SOD2, Bcl-2, and I*κ*B-*α* in the H_2_O_2_ treatment group were significantly decreased compared with the H_2_O_2_+escin group, and the Bax, TNF-*α*, IL-1*β*, p65, and I*κ*K*α* protein expressions were greatly higher than those in the H_2_O_2_+escin group. And the results of PCR were also consistent with those. TUNEL-positive cells also decreased significantly when treated with escin. Flow cytometry showed that the percentage of apoptotic cells decreased greatly after treatment of escin. Through IL-1*β* immunofluorescence, the fluorescence intensity of the H_2_O_2_ treatment group was greatly higher compared with that of the control group, but escin reversed this effect.

**Conclusions:**

These results indicated that escin inhibits H_2_O_2_-induced H9c2 cell apoptosis, oxidative stress, and inflammatory responses via the NF-*κ*B signaling pathway.

## 1. Introduction

Myocardial infarction is a serious cardiovascular disease in the world. It is mainly due to coronary atherosclerosis which leads to narrowing of the lumen and causes damage to the myocardium innervated by blocked blood vessels [1]. Due to ischemia and hypoxia, myocardial infarction produces excessive ROS, which destroys surrounding myocardial tissue and further causes apoptosis of cardiomyocytes [2]. It is well known that cardiomyocytes are nonrenewable, and oxidative stress and apoptosis of the myocardium can lead to the death of some myocardium, eventually leading to deterioration of heart function and even death. The existing treatments are mainly to achieve recanalization of blood vessels but cannot reverse the apoptosis of cardiomyocytes, and myocardial ischemia and reperfusion may aggravate oxidative stress [3]. Therefore, new treatments are urgently needed to inhibit myocardial oxidative stress and apoptosis in the early stages of myocardial infarction.

Escin is a pentacyclic triterpenoid saponin extracted from the dried mature seeds of the horse chestnut [4]. At present, there are many studies describing the role of escin in various histopathophysiology, including ovary, nervous system, and skin. Many people have also studied its role in tumors. Selvakumar et al. [5] believed that escin plays an antiapoptotic and antioxidative stress in Parkinson's disease and can improve the motor function of patients. Wang et al. [6] believed that escin can play an antioxidative stress on retinal pigment epithelial cells. Cheng et al. [7] suggested that escin can regulate apoptosis in ovarian cancer. However, the effect of escin on cardiomyocytes has not been studied.

In this study, H_2_O_2_-induced myocardial cell injury model was adopted to investigate whether escin can play an antioxidative, antiapoptotic, and anti-inflammatory role and its potential mechanism of action. Our results showed that escin may provide a potential new treatment for MI.

## 2. Materials and Methods

### 2.1. Cell Culture

Dulbecco's Modified Eagle's Medium (MCE, Nanjing, China) complemented with 10% fetal bovine serum (FBS) (MCE, Nanjing, China) was used to culture H9c2 cells in 37 degrees Celsius incubator under 5% CO_2_. When the cells grew to about 80% confluence, they began to be plated and treated with H_2_O_2_ (100 *μ*M) for 4 hours and escin (10 *μ*M) for 24 hours.

### 2.2. Drug Preparation

Escin was purchased from Tianpu Biochemical Pharmaceutical in Guangdong (Guangzhou, China). The stock solution was stored in a refrigerator at -20 degrees Celsius.

### 2.3. RNA Extraction and Real-Time Polymerase Chain Reaction (PCR)

TRIzol reagent (MCE, Nanjing, China) was utilized to extract the total RNA of H9c2 cells. 0.5 ml of TRIzol was added into each well of a 24-well plates, and the liquid was transferred to the EP tube after grinding. Then, chloroform was added in 1/5 times of the amount of TRIzol. We let the eppendorf (EP) tubes shake, and let it stand for 5 minutes. After that, the EP tubes were centrifuged with a centrifugal force of 12000 × g for about 20 minutes at 4°C. We aspirated the upper aqueous phase of the mixture obtained by centrifugation and added an equal amount of isopropanol, mixed it, and placed it at 4°C for 15 minutes. Then, we centrifuged the mixture at 4°C for 15 minutes with a centrifugal force of 12000 × g, after which the supernatant was discarded and 1 mL of a 75% ethanol solution was added. The solution was centrifuged at 4°C for 5-10 minutes with a centrifugal force of 7500 × g, after which the supernatant was discarded and dried at room temperature, and 20 *μ*L of ribonuclease free water was added. Finally, we used NanoDropTM 8000 to measure RNA concentration.

Reverse transcription was performed using reverse transcriptase kit (MCE, Nanjing, China). Real-time PCR was performed by using Prism 7900 System. We used a 10 *μ*L reaction system in accordance with the protocol. GAPDH (MCE, Nanjing, China) was used to standardize the data. All the primers are listed in Table 1.

### 2.4. Western Blot

The total protein of the cells placed in a 6-well plate was extracted by a protein extraction kit [8]. The protein concentration was measured by the bicinchoninic acid (BCA) method (Pierce, Rockford, IL, USA). Then, the loading buffer was added to the total protein and boiled the mixture for 7 minutes. After that, we took the same amount of total protein and performed electrophoresis with 10% sodium dodecyl sulphate-polyacrylamide gel electrophoresis (SDS-PAGE). Then, we transferred the electrophoresed protein to the polyvinylidene fluoride (PVDF, EpiZyme, Shanghai, China) membrane. 5% concentration of skim milk was prepared with Tween-20 (TBST) to block the nonspecific antigens of the protein bands. After 2 hours, the primary antibodies were used to incubate the bands overnight (SOD1, Abcam, Cambridge, MA, USA, Rabbit, 1 : 2000; SOD2, Abcam, Cambridge, MA, USA, Rabbit, 1 : 2000; Bcl-2, Abcam, Cambridge, MA, USA, Mouse, 1 : 1000; Bax, Abcam, Cambridge, MA, USA, Mouse, 1 : 1000; TNF-*α*, Abcam, Cambridge, MA, USA, Rabbit, 1 : 1000; IL-1*β*, Abcam, Cambridge, MA, USA, Rabbit, 1 : 5000; p65, Abcam, Cambridge, MA, USA, Rabbit, 1 : 1000; I*κ*K*α*, Abcam, Cambridge, MA, USA, Rabbit, 1 : 2000; I*κ*B-*α*, Abcam, Cambridge, MA, USA, Rabbit, 1 : 2000; and GAPDH, Proteintech, Rosemont, IL, USA, 1 : 3000), followed by incubation with secondary antibodies for 1.5 hours. After the bands were washed 3 times for 30 minutes, the protein bands were exposed by the Image Lab™ Software.

### 2.5. ROS Quantification

Quantification of ROS was performed using the DHR-ROS test kit (KeyGen, Shanghai, China) in accordance with the manufacturer's protocol.

### 2.6. Superoxide Dismutase (SOD) Activity Assay

We lysed the H9c2 cells with lysate and collected and centrifuged to remove the supernatant. Detection of SOD levels in cells was performed by the SOD assay kit (KeyGen, Shanghai, China) in accordance with the protocol.

### 2.7. TUNEL Staining

TUNEL kit (Roche, Basel, Switzerland) was used as instructed by the manufacturer to detect the apoptotic cells. Briefly, the H9c2 cells were fixed with 4% paraformaldehyde for 20 minutes at room temperature and then treated with Triton X-100 for another 20 minutes. After that, the cells were treated with 150 *μ*l TUNEL reaction solution at 37°C for 60 minutes. And the nucleus was stained with DAPI (Beyotime Biotechnology, Shanghai, China). TUNEL staining was shown by a Confocal Laser Scanning Microscope (CLSM).

### 2.8. Cell Counting Kit-8 (CCK-8) Assay

We placed H9c2 cells in a 96-well plate. After H_2_O_2_ and escin treatment, the viability of different groups of cells was examined by CCK-8 assay (Dojindo, Kumamoto, Japan) to explore the effect of escin on H9c2 cells. The absorbance at 450 nm was detected using a microplate reader.

### 2.9. Flow Cytometry

H9c2 cells were treated with H_2_O_2_ and escin as described above. The supernatant was then collected, and adherent cells were collected after digestion with trypsin. We centrifuged the cell suspension with a centrifugal force of 200 × g for 5 minutes, discarded the supernatant, washed with PBS, and centrifuged again in the same manner, repeating twice. Then, we resuspended the cells with 100 *μ*L of binding buffer after discarding the supernatant. After that, we added 5 *μ*L of Annexin V-FITc (KeyGen, Shanghai, China) and 5 *μ*L of propidium iodide (KeyGen, Shanghai, China) in per tube in the dark. Finally, the apoptotic H9c2 cells were detected using flow cytometry.

### 2.10. IL-1*β* Immunofluorescence

H9c2 cells were placed in a 24-well plate, fixed with 4% paraformaldehyde after the above treatment, then added goat serum, and incubated for 1 hour. After that, an appropriate amount of primary antibody IL-1*β* was added and incubated overnight at 4 degrees Celsius. On the second day, we added the corresponding fluorescent secondary antibody and incubated them in the dark for 1 hour. 4′,6-Diamidino-2-phenylindole (DAPI) was also added to stain the nucleus. Finally, it was observed by a Confocal Laser Scanning Microscope (CLSM).

### 2.11. Statistics Analysis

Measurement data is expressed as *χ* ± *s*. Differences between two groups were analyzed by using the Student *t*-test. Comparison between multiple groups was done using one-way ANOVA test followed by post hoc test (Least Significant Difference). Least Significant Difference (LSD) test or Student-Newman-Keuls (SNK) test was used for pairwise comparison under the condition of homogeneity of variance. Test level *α* = 0.05. All experiments were repeated 3 times.

## 3. Results

### 3.1. Escin Protected H_2_O_2_-Induced H9c2 Cell Injury

**Figure 1:**
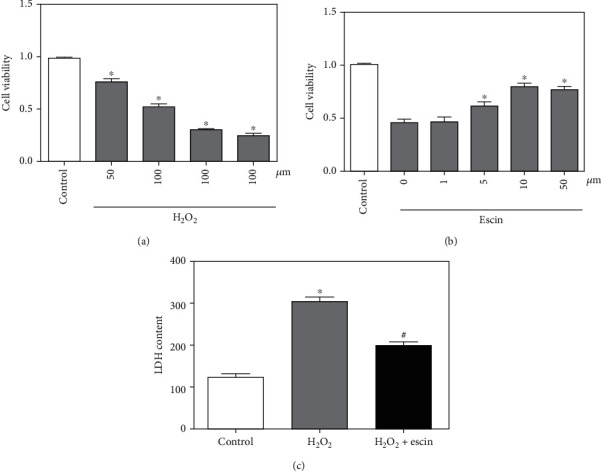
Escin protected H9c2 cells from damage. (a) CCK-8 assay showed viability of H9c2 cells at different concentrations of H_2_O_2 (_^∗^*p* < 0.05 vs. control, *n* = 3). (b) CCK-8 assay showed cell viability after addition of different concentrations of escin in H_2_O_2_-treated H9c2 cells (^∗^*p* < 0.05 vs. 0 *μ*M, *n* = 3). (c) The LDH content increased significantly in the H_2_O_2_ treatment group and decreased significantly in the H_2_O_2_+escin group (^∗^*p* < 0.05 vs. control; ^#^*p* < 0.05 vs. H_2_O_2_, *n* = 3).

To measure the appropriate H_2_O_2_ concentration for H9c2 cells, H9c2 cells were treated with different concentrations of H_2_O_2_ for 4 hours. As shown in Figure 1(a), when H9c2 cells were treated with 100 *μ*M of H_2_O_2_, cell viability was suppressed by about 50% by CCK-8 assay, so we selected 100 *μ*M of H_2_O_2_ for subsequent studies. Next, in order to determine the optimal dosing concentration, we treated H_2_O_2_-treated H9c2 cells with different concentrations of escin for 24 hours. We measured cell viability using CCK-8 assay, and the highest cell viability was obtained when H9c2 cells were treated with 10 *μ*M of escin (Figure 1(b)). After that, we collected the supernatant of H9c2 cells and detected them with the LDH kit. The results showed that escin significantly inhibited H_2_O_2_-mediated LDH elevation (Figure 1(c)). These results indicated that escin can act directly on H9c2 cells and reduced the damage caused by H_2_O_2_.

### 3.2. Escin Inhibited H_2_O_2_-Induced Oxidative Stress in H9c2 Cells

**Figure 2:**
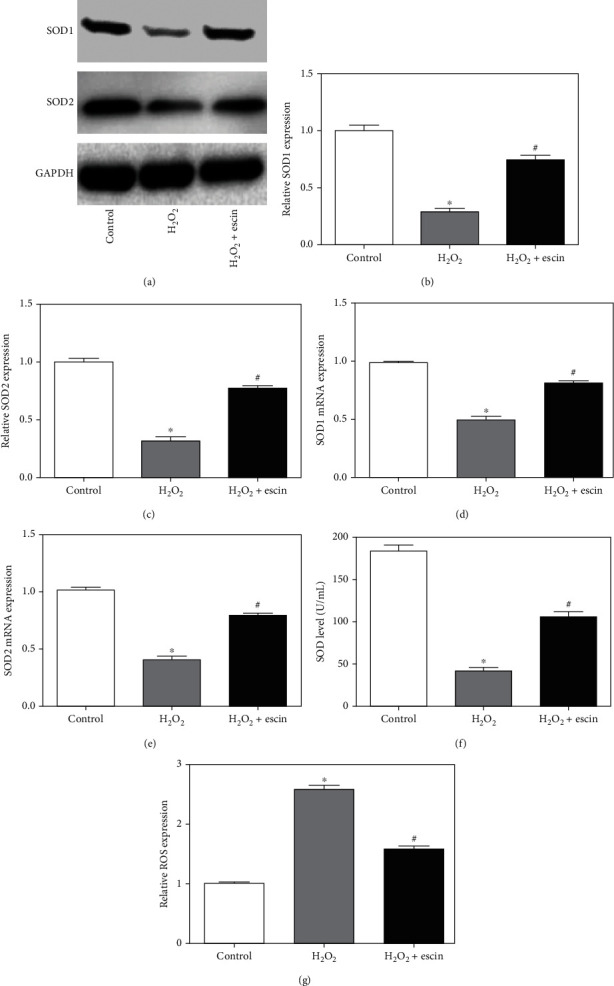
Escin inhibited oxidative stress in H9c2 cells. (a) The expression of SOD1 and SOD2 in the H_2_O_2_ treatment group decreased significantly, and the expression of SOD1 and SOD2 increased in the H_2_O_2_+escin group. (b) Statistical results of protein level of SOD1 (^∗^*p* < 0.05 vs. control; ^#^*p* < 0.05 vs. H_2_O_2_, *n* = 3). (c) Statistical results of protein level of SOD2 (^∗^*p* < 0.05 vs. control; ^#^*p* < 0.05 vs. H_2_O_2_, *n* = 3). (d) The expression of SOD1 mRNA in the H_2_O_2_ treatment group decreased significantly, and the expression increased in the H_2_O_2_+escin group (^∗^*p* < 0.05 vs. control; ^#^*p* < 0.05 vs. H_2_O_2_, *n* = 3). (e) The expression of SOD2 mRNA in the H_2_O_2_ treatment group decreased significantly, and the expression increased in the H_2_O_2_+escin group (^∗^*p* < 0.05 vs. control; ^#^*p* < 0.05 vs. H_2_O_2_, *n* = 3). (f) SOD activity assay showed that H_2_O_2_ can significantly reduce SOD levels, while escin can reverse SOD levels (^∗^*p* < 0.05 vs. control; ^#^*p* < 0.05 vs. H_2_O_2_, *n* = 3). (g) The expression of ROS increased in the H_2_O_2_ treatment group but decreased significantly in the H_2_O_2_+escin group (^∗^*p* < 0.05 vs. control; ^#^*p* < 0.05 vs. H_2_O_2_, *n* = 3).

We used Western blot to detect the expression levels of SOD1 protein and SOD2 protein (Figure 2(a)). In the H_2_O_2_ treatment group, the expression levels of SOD1 and SOD2 were greatly decreased compared to the control group, while the treatment with escin greatly increased the expression levels of SOD1 and SOD2 (Figures 2(b) and 2(c)). Then, we used real-time PCR to detect the expression levels of SOD1 mRNA and SOD2 mRNA, which were consistent with the results of Western blot (Figures 2(d) and 2(e)). At the same time, the SOD activity assay was used to detect the level of SOD. The results showed that the addition of escin can significantly reverse the decrease of H_2_O_2_-mediated SOD levels (Figure 2(f)). Finally, we tested the level of ROS production by DHR dye assay. We can see from Figure 2(g) that the treatment of H_2_O_2_ increased the level of ROS production, while in the H_2_O_2_+escin group, the level of ROS production decreased significantly. These results demonstrated that escin can inhibit oxidative stress in H9c2 cells treated with H_2_O_2_.

### 3.3. Escin Inhibited H_2_O_2_-Induced Apoptosis in H9c2 Cells

**Figure 3:**
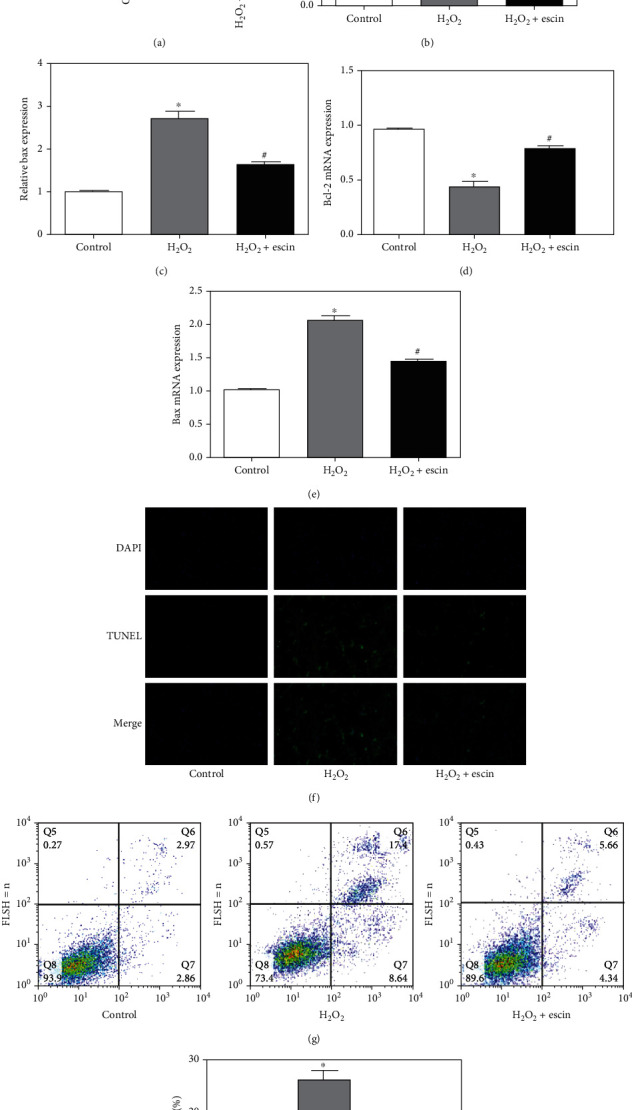
Escin inhibited apoptosis of H9c2 cells. (a) The expression of Bcl-2 in the H_2_O_2_ treatment group decreased significantly but increased in the H_2_O_2_+escin group. Bax expression was opposite to Bcl-2. (b) Statistical results of protein level of Bcl-2 (^∗^*p* < 0.05 vs. control; ^#^*p* < 0.05 vs. H_2_O_2_, *n* = 3). (c) Statistical results of expression of Bax (^∗^*p* < 0.05 vs. control; ^#^*p* < 0.05 vs. H_2_O_2_, *n* = 3). (d) The expression of Bcl-2 mRNA was consistent with the Bcl-2 protein (^∗^*p* < 0.05 vs. control; ^#^*p* < 0.05 vs. H_2_O_2_, *n* = 3). (e) The expression of Bax mRNA was consistent with the Bax protein (^∗^*p* < 0.05 vs. control; ^#^*p* < 0.05 vs. H_2_O_2_, *n* = 3). (f) TUNEL staining showed that escin can obviously reduce the increase of H9c2 cell apoptosis caused by H_2_O_2_ (magnification: ×400). (g) The apoptotic rate of the H_2_O_2_ treatment group increased and decreased in the H_2_O_2_+escin group. (h) Statistical results of apoptotic rate of H9c2 cells (^∗^*p* < 0.05 vs. control; ^#^*p* < 0.05 vs. H_2_O_2_, *n* = 3).

First, we examined the expression of Bcl-2 and Bax proteins by Western blot (Figure 3(a)). It can be seen that the expression of Bcl-2 in the H_2_O_2_ treatment group was greatly decreased compared with the control group, while the level of Bax was greatly increased. After using escin, the level of Bcl-2 was increased and the level of Bax was decreased (Figures 3(b) and 3(c)). Similarly, we detected the levels of Bcl-2 and Bax mRNA using real-time PCR, which was consistent with the results of Western blot (Figures 3(d) and 3(e)). To further demonstrate the antiapoptotic effect of escin, we used TUNEL staining to detect the level of TUNEL-positive cells. It can be seen that the TUNEL-positive cells in the H_2_O_2_+escin group were greatly reduced compared to the H_2_O_2_ treatment group (Figure 3(f)). We also used flow cytometry to detect the effect of escin on H9c2 cells treated with H_2_O_2_. We can see that escin can significantly reduce the apoptotic rate of H_2_O_2_-treated H9c2 cells (Figures 3(g) and 3(h)). These results indicated that escin can inhibit H_2_O_2_-mediated apoptosis of H9c2 cells.

### 3.4. Escin Inhibited H_2_O_2_-Induced Inflammation of H9c2 Cells

**Figure 4:**
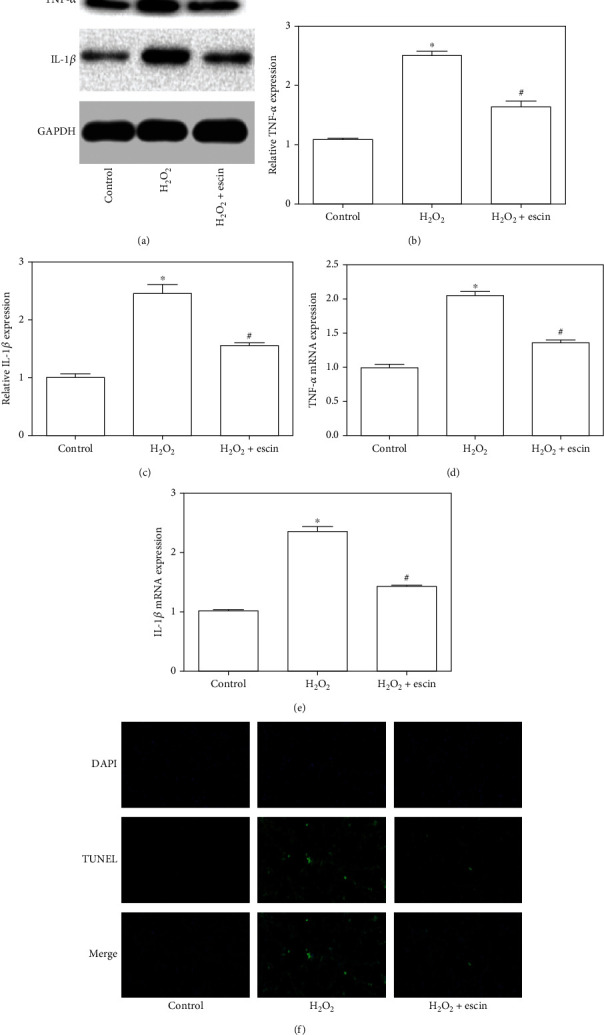
Escin inhibited inflammation of H9c2 cells. (a) The expression of IL-1*β* and TNF-*α* in the H_2_O_2_ treatment group increased significantly but decreased in the H_2_O_2_+escin group. (b) Statistical results of expression of TNF-*α* (^∗^*p* < 0.05 vs. control; ^#^*p* < 0.05 vs. H_2_O_2_, *n* = 3). (c) Statistical results of expression of IL-1*β* (^∗^*p* < 0.05 vs. control; ^#^*p* < 0.05 vs. H_2_O_2_, *n* = 3). (d) TNF-*α* mRNA expression was similar to the results of Western blot (^∗^*p* < 0.05 vs. control; ^#^*p* < 0.05 vs. H_2_O_2_, *n* = 3). (e) IL-1*β* mRNA expression was also similar to the results of Western blot (^∗^*p* < 0.05 vs. control; ^#^*p* < 0.05 vs. H_2_O_2_, *n* = 3). (f) Immunofluorescence showed that escin significantly reduced H_2_O_2_-mediated elevation of IL-1*β* (magnification: ×400).

The expression of IL-1*β* and TNF-*α* was examined by Western blotting (Figure 4(a)). We can see that H_2_O_2_ significantly induced the increase of IL-1*β* and TNF-*α*, while the escin inhibited their increase (Figures 4(b) and 4(c)). Similar results were obtained for mRNA levels (Figures 4(d) and 4(e)). Immunofluorescence showed that IL-1*β* expression in the H_2_O_2_+escin group was significantly decreased compared with the H_2_O_2_ treatment group (Figure 4(f)). These results showed that escin can inhibit the inflammation of H9c2 cells induced by H_2_O_2_.

### 3.5. Escin Inhibited the NF-*κ*B Pathway

**Figure 5:**
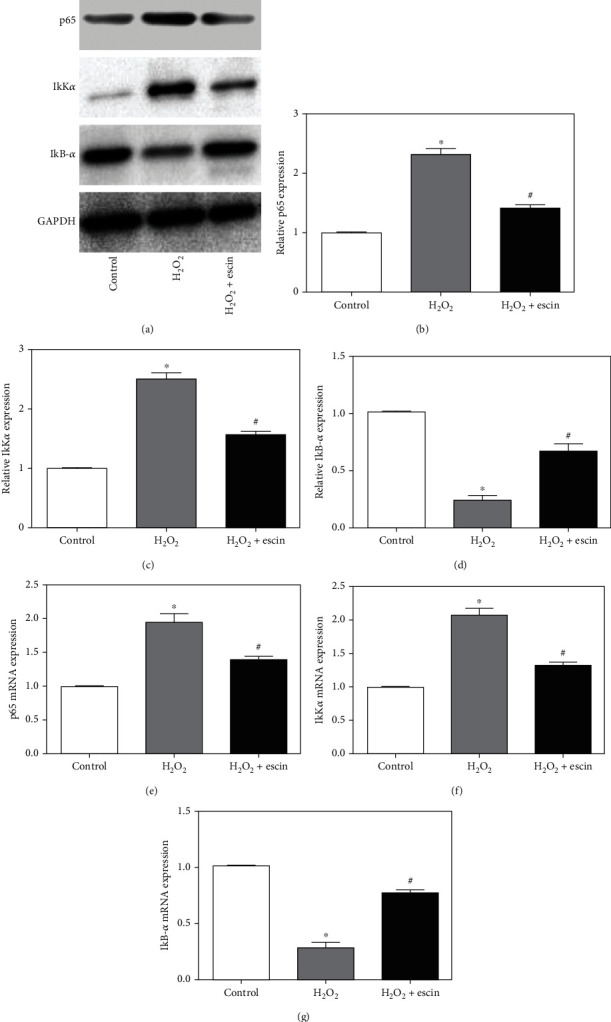
Escin inhibited the NF-*κ*B pathway. (a) In the H_2_O_2_ treatment group, the expression of p65 and I*κ*K*α* increased, and the expression of I*κ*B-*α* decreased. In the H_2_O_2_+escin group, the expression of p65 and I*κ*K*α* decreased, and the expression of I*κ*B-*α* increased. (b) Statistical results of protein level of p65 (^∗^*p* < 0.05 vs. control; ^#^*p* < 0.05 vs. H_2_O_2_, *n* = 3). (c) Statistical results of protein level of I*κ*K*α* (^∗^*p* < 0.05 vs. control; ^#^*p* < 0.05 vs. H_2_O_2_, *n* = 3). (d) Statistical results of protein level of I*κ*B-*α* (^∗^*p* < 0.05 vs. control; ^#^*p* < 0.05 vs. H_2_O_2_, *n* = 3). (e) Expression of p65 mRNA in three groups (^∗^*p* < 0.05 vs. control; ^#^*p* < 0.05 vs. H_2_O_2_, *n* = 3). (f) Expression of I*κ*K*α* mRNA in three groups (^∗^*p* < 0.05 vs. control; ^#^*p* < 0.05 vs. H_2_O_2_, *n* = 3). (g) Expression of I*κ*B-*α* mRNA was consistent with the results of Western blot (^∗^*p* < 0.05 vs. control; ^#^*p* < 0.05 vs. H_2_O_2_, *n* = 3).

The NF-*κ*B pathway plays a very important role in regulating apoptosis and oxidative stress, so we examined the NF-*κ*B pathway in different treatment groups by Western blot (Figure 5(a)). We can see from Figures 5(b)–5(d) that the expression of p65 and I*κ*B kinase *α* (I*κ*K*α*) in the H_2_O_2_ treatment group was significantly increased, while the expression of inflammatory inhibitor NF-*κ*B*α* (I*κ*B-*α*) was greatly decreased, compared with the control group. After treatment with escin, the expression of p65 and I*κ*K*α* was decreased, while the expression level of I*κ*B-*α* increased compared to the H_2_O_2_ treatment group. And the results of the real-time PCR matched the results of the Western blot (Figures 5(e)–5(g)). These results suggested that escin can inhibit the NF-*κ*B pathway.

## 4. Discussion

In this present study, we revealed for the first time the role of escin in MI. H_2_O_2_ treatment successfully induced oxidative stress, inflammation, and apoptosis of H9c2 cardiomyocytes. However, escin can reverse the damage of H9c2 cells caused by H_2_O_2_. This protective effect was achieved at least in part by targeting the NF-*κ*B pathway.

AMI is a serious health problem in the world with a high mortality rate [9]. Current treatments can reconstitute the blood supply to the ischemic areas [10]. However, these treatments do not fully restore the apoptosis and oxidative stress of some cardiomyocytes. Previous studies have shown that in AMI, the myocardial ischemia site produces a large amount of ROS due to oxidative stress, which causes damage to the myocardial cell membrane, and at the same time produces a large number of inflammatory factors, causing apoptosis of cardiomyocytes [11]. Therefore, in addition to rebuilding the blood supply in the ischemic areas, treatments such as antioxidative stress, antiapoptosis, and anti-inflammatory are also essential.

Many studies have previously reported antioxidative, antiapoptotic, and anti-inflammatory effects of escin in other diseases. So, we want to verify whether escin has a similar effect in MI. We first tested the cell viability of H9c2 cells treated with H_2_O_2_ and found that escin did increase the cell viability. Then, we examined the expression of oxidative stress-related proteins, apoptosis-related proteins, and some inflammatory factors and also detected the corresponding mRNA expression. The final result confirmed our hypothesis that escin can inhibit oxidative stress, apoptosis, and inflammation in H9c2 cells, thereby increasing cell viability. This also provided a potential treatment for MI.

Studies have shown that MI involves multiple signaling pathways such as Rho/ROCK pathway, MAPK pathway, and NF-*κ*B pathway [12]. Among them, NF-*κ*B pathway is a classical signaling pathway that has an important influence on inflammation, apoptosis, and oxidative stress. Xiong et al. [13] believed that quercetin acts to treat periodontitis through NF-*κ*B signaling pathway. Yi et al. [14] suggested that inhibition of NF-*κ*B signaling pathway promoted autophagy and inhibited apoptosis and inflammation in nucleus pulposus cells. By detecting the expression of p65, I*κ*K*α*, I*κ*B-*α* protein, and mRNA in different treatment groups, our study also confirmed that escin can inhibit cardiomyocyte apoptosis, inflammation, and oxidative stress through the NF-*κ*B pathway.

In summary, our study suggests that the effect of escin on MI is primarily due to the inhibition of the NF-*κ*B signaling pathway. This finding is of great help to the clinical treatment of MI.

## 5. Conclusions

Escin inhibits the NF-*κ*B pathway and thus inhibits oxidative stress, apoptosis, and inflammation induced by H_2_O_2_ in H9c2 cells. Therefore, escin may provide a new treatment for MI.

## Figures and Tables

**Table 1:** Real-time PCR primers.

Gene name	Forward (5′>3′)	Reverse (5′>3′)
Bax	CAGTTGAAGTTGCCATCAGC	CAGTTGAAGTTACCATCAGC
Bcl-2	GACTGAGTACCTGAACCGGCATC	CTGAGCAGCGTCTTCAGAGACA
SOD1	GGTGAACCAGTTGTGTTGTC	CCGTCCTTTCCAGCAGTC
SOD2	CAGACCTGCCTTACGACTATGG	CTCGGTGGCGTTGAGATTGTT
IL-1*β*	GCAACTGTTCCTGAACTCAACT	ATCTTTTGGGGTCCGTCAACT
TNF-*α*	CCTCTCTCTAATCAGCCCTCTG	GAGGACCTGGGAGTAGATGAG
I*κ*K*α*	GTCAGGACCGTGTTCTCAAGG	GCTTCTTTGATGTTACTGAGGGC
I*κ*B-*α*	GGATCTAGCAGCTACGTACG	TTAGGACCTGACGTAACACG
p65	ACTGCCGGGATGGCTACTAT	TCTGGATTCGCTGGCTAATGG
GAPDH	ACAACTTTGGTATCGTGGAAGG	GCCATCACGCCACAGTTTC

RT-PCR: quantitative reverse-transcription polymerase chain reaction.

## Data Availability

The datasets used and analyzed during the current study are available from the corresponding author on reasonable request.
